# Predictive Factors Increasing the Risk of Radiation Toxicity in Patients with Early Breast Cancer

**DOI:** 10.31557/APJCP.2021.22.1.145

**Published:** 2021-01

**Authors:** Asmaa A Abdeltawab, Samia A Ali, Hanan G Mostafa, Mohamed A Hassan

**Affiliations:** *Department of Clinical Oncology, Faculty of Medicine, Assiut University, Assiut, Egypt. *

**Keywords:** Breast cancer, conventional radiotherapy, normal tissue toxicity, risk factors

## Abstract

**Objectives:**

Radiation induces adverse events on healthy tissues which may be augmented by certain factors. This study aimed to assess patients; tumor and treatment-related factors which increase the risk of radiation-induced toxicity in breast cancer patients.

**Methods:**

This prospective study included postmenopausal early breast cancer patients treated at the clinical oncology department, Assiut University, Egypt between January 2015 and December 2018. Patients treated with mastectomy followed by conventional radiotherapy (25x 2 Gy) and either concurrent or sequential letrozole. Acute and late radiation toxicity was scored according to EORTC/RTOG and risk factors were analyzed.

**Results:**

A total of 75 patients were included in the study. After a median follow-up of 24 months, 12 patients had > grade 2 acute dermatitis, 5 patients had > grade 2 cardiac toxicity and 3 patients had > grade 2 lung toxicity. Multivariate analysis revealed that trastuzumab use was associated with a decrease risk of acute dermatitis (p= 0.01) but boost irradiation was significantly associated with increased risk of acute dermatitis (p= 0.01). Late toxicity > grade 2 was observed in 6 patients, 14 patients, and 2 patients for skin, heart, and lung respectively.

**Conclusion:**

The use of boost irradiation was associated with increased risk of acute dermatitis, in the contrary; the use of trastuzumab seemed to be protective as observed in this study.

## Materials and Methods

This was a prospective study of postmenopausal patients with early breast cancer who received adjuvant radiotherapy (RT) at the clinical oncology department (Assiut University Hospital, Assiut, Egypt) between January 2015 and December 2018. Only patients with operable breast cancer were selected, and they should be hormone receptor positive. All eligible patients included in this study were randomized by a simple randomization manner in a 1:1 ratio before receiving either sequential or concurrent letrozole (2.5 mg daily orally for 5 years) with RT. Patients with HER2-neu positive disease were given trastuzumab treatment after RT. Radiation was either to the whole breast (after breast conservative surgery) and chest wall after mastectomy, regional lymph nodes like supraclavicular or internal mammary lymph node, or both were irradiated when indicated. Opposed medical and lateral tangential fields and wedges were used to compensate for missing tissues in the irradiation of the tumor bed and chest wall. Direct portal for supraclavicular lymph node irradiation and wide tangential fields were used to treat internal mammary lymph nodes. Two-dimensional planning was used at a dose of 50 Gy in 25 fractions over 5 weeks (2 Gy per fraction). A single anterior photon field for the supraclavicular region to a total dose of 50 Gy was applied. Boost was given either by photon beam or electron beam energy 9–18 MeV at a dose of 10 Gy in a 2 GY per fraction for a week after BCS was used according to guidelines. A teletherapy Cobalt-60 machine (Nordon, Elite 100) and Linear accelerator (Primus, Siemens) were utilized to treat the patients in the study with RT. Early and late toxicities of radiation to the lung, heart, and skin were analyzed prospectively using the Radiation Therapy Oncology Group and the European Organization for Research and Treatment of Cancer (RTOG/EORTC) (Cox et al., 1995). Early radiation toxicity was defined as occurring in 90 days while late radiation occurred after 90 days.

The protocol was approved by the local institutional boards and ethics committee of Assiut University. All patients provided written informed consent.

*Statistical analysis*

Numerical variables are presented as the mean ± standard deviation and categorical variables are presented as count and percent. Student’s t-test was used to compare the numerical variables. Pearson’s* χ*^2^ test was used to compare independent categorical variables. Multivariate logistic regression analysis was performed to identify the predictors of skin, cardiac, and pulmonary toxicity. A p-value < 0.05 was regarded as statistically significant. Statistical analysis was performed using SPSS software version 23.0.

## Results

The mean age of all 75 patients was 59.47 years (range, 44-80 years). The body mass index (BMI) ≥25 Kg/m2 was detected in 90.7% of patients. Hypertension and diabetes were diagnosed in 36% and 24% of patients respectively. There was no heart disease in all investigated patients. There was no heart disease in all investigated patients. All patients had hormonal receptor-positive disease but 20% of them had a positive HER2-neu disease. 

As regards treatment of the studied patients, nodal irradiation was administered in 10.7% of patients, and boost radiotherapy was applied in 18.7% of them. 74.7% of studied patients received anthracycline only based chemotherapy, 18.7% received taxane after anthracycline chemotherapy, while 6.6% only did not receive any adjuvant chemotherapy. Trastuzumab was used for the treatment of 20% of patients. 

In terms of the influencing factors for acute skin dermatitis, univariate analyses showed a significant difference between patients with ≤ G2 acute skin toxicity (33.3%) and those with > G2 toxicity (41.7%) who received trastuzumab therapy in comparison to those without acute skin toxicity (13%). Also, patients who received boost RT had a significantly higher >G2 acute skin toxicity (58.3%) than patients with no toxicity (3.7%) as shown in Table 1.

With multivariate regression analysis showed that use of trastuzumab (odds ratio= 4.53, 95%CI= 1.27-16.14, P= 0.01) and boost irradiation (odds ratio= 4.61, 95%CI= 1.33-15.93, P= 0.01) are predictors for acute skin toxicity.

Table 2 shows the association between the characteristics of patients and therapy-related risk factors with late skin toxicity. It was noticed that different grades of late skin toxicity had insignificant differences regarding baseline data except patients with >G2 late skin toxicity who received boost irradiation.

Multivariate regression analysis, boost irradiation wasn’t predictor for late skin toxicity (odds ratio= 1.68, 95%CI= 0.49-5.67, P= 0.40).

None of enrolled patients had late cardiac toxicity ≤ G2 while 14 patients from all studied patients had late cardiac toxicity > G2. It was noticed that patients with late cardiac toxicity > G2 had insignificant differences as regarding baseline demographic data with exception of significantly higher frequency of hypertension among patients with late cardiac toxicity > G2 10 (71.4%) vs. 17 (27.9%); P< 0.001) as shown in table 3.

Multivariate regression analysis, hypertension wasn’t a predictor for late cardiac toxicity (odds ratio= 2.05, 95%CI= 0.58-7.18, P= 0.26). 

It was noticed that patients with different grades of late pulmonary toxicity had insignificant factors influencing their toxicity. 

**Table 1:** Acute Skin Toxicity in Association with Patient- and Therapy-Related Characteristics

	Acute skin toxicity			
Data	No (n= 54)	≤ G2 (n= 9)	> G2 (n= 12)	P value
Hormonal treatment				
Sequential	32 (59.3%)	4 (44.4%)	4 (33.3%)	0.22
Concurrent	22 (40.7%)	5 (55.6%)	8 (66.7%)	
BMI				
< 25 kg/m^2^	5 (9.3%)	0	2 (16.7%)	0.43
≥ 25 kg/m^2^	49 (90.7%)	9 (100%)	10 (83.3%)	
DM				
Yes	13 (24.1%)	1 (11.1%)	4 (33.3%)	0.49
No	41 (75.9%)	8 (88.9%)	8 (66.7%)	
HTN				
Yes	21 (38.9%)	4 (44.4%)	2 (16.7%)	0.29
No	33 (61.1%)	5 (55.6%)	10 (83.3%)	
Tumor side				
Left	28 (51.9%)	3 (33.3%)	6 (50%)	0.58
Right	26 (48.1%)	6 (66.7%)	6 (50%)	
Chemotherapy				
Anthracycline with taxane	8 (14.8%)	3 (33.3%)	3 (25%)	0.44
Anthracycline- based	41 (75.9%)	6 (66.7%)	9 (75%)	
No	5 (9.3%)	0	0	
Trastuzumab treatment				
Yes	7 (13%)	3 (33.3%)	5 (41.7%)	0.03
No	47 (87%)	6 (66.7%)	7 (58.3%)	
Nodal irradiation				
No	6 (11.1%)	1 (11.1%)	1 (8.3%)	0.96
Yes	48 (88.9%)	8 (88.9%)	11 (91.7%)	
Boost irradiation				
Yes	2 (3.7%)	5 (55.6%)	7 (58.3%)	0.01
No	52 (92.3%)	4 (44.4%)	5 (41.7%)	

Data expressed as frequency (percentage). P value was significant if < 0.05 and is shown in bold. BMI, body mass index; DM, diabetes mellitus; HTN, hypertension.

**Table 2:** Late Skin Toxicity in Association with Demographic Data

	Late skin toxicity			
Data	No (n= 62)	≤ G2 (n= 6)	> G2 (n= 7)	P value
Hormonal treatment				
Sequential	35 (56.5%)	2 (33.3%)	3 (42.9%)	0.46
Concurrent	27 (43.5%)	4 (66.7%)	4 (57.1%)	
BMI				
< 25 kg/m^2^	6 (9.7%)	1 (16.7%)	0	0.57
≥ 25 kg/m^2^	56 (90.3%)	5 (83.3%)	7 (100%)	
DM				
Yes	16 (25.8%)	1 (16.7%)	1 (14.3%)	0.72
No	46 (74.2%)	5 (83.3%)	6 (85.7%)	
HTN				
Yes	21 (33.9%)	3 (50%)	3 (42.9%)	0.67
No	41 (66.1%)	3 (50%)	4 (57.1%)	
Tumor side				
Left	32 (51.6%)	2 (33.3%)	3 (42.9%)	0.65
Right	30 (48.4%)	4 (66.7%)	4 (57.1%)	
Chemotherapy				
Anthracycline with taxane	11 (17.7%)	2 (33.3%)	1 (14.3%)	0.73
Anthracycline -based	46 (74.2%)	4 (66.7%)	6 (85.7%)	
No	5 (8.1%)	0	0	
Trastuzumab treatment				
Yes	12 (19.4%)	0	3 (42.9%)	0.14
No	50 (80.6%)	6 (100%)	4 (57.1%)	
Nodal irradiation				
No	6 (9.7%)	1 (16.7%)	1 (14.3%)	0.24
Yes	56 (90.3%)	5 (83.3%)	6 (85.7%)	
Boost irradiation				
Yes	3 (4.8%)	4 (66.7%)	7 (100%)	< 0.001
No	59 (95.2%)	2 (33.3%)	0	

Data expressed as frequency (percentage). P value was significant if < 0.05. BMI: body mass index; DM, diabetes mellitus; HTN, hypertension

**Table 3:** Association between Patient- and Therapy-Related Variables and Late Cardiac Toxicity

	Late cardiac toxicity		
Data	No (n= 61)	> G2 (n= 14)	P value
Hormonal treatment			
Sequential	32 (52.5%)	8 (57.1%)	0.49
Concurrent	29 (47.5%)	6 (42.9%)	
BMI			
< 25 kg/m^2^	7 (11.5%)	0	0.22
≥ 25 kg/m^2^	54 (88.5%)	14 (100%)	
DM			
Yes	13 (21.3%)	5 (35.7%)	0.21
No	48 (78.7%)	9 (64.3%)	
HTN			
Yes	17 (27.9%)	10 (71.4%)	< 0.001
No	44 (72.1%)	4 (28.6%)	
Tumor side			
Left	29 (47.5%)	8 (57.1%)	0.36
Right	32 (52.2%)	6 (42.9%)	
Chemotherapy			
Anthracycline with taxane	10 (16.4%)	4 (28.6%)	0.56
Anthracycline-based	47 (77%)	9 (64.3%)	
No	4 (6.6%)	1 (7.1%)	
Trastuzumab treatment			
Yes	10 (16.4%)	5 (35.7%)	0.1
No	51 (83.6%)	9 (64.3%)	
Nodal irradiation			
No	7 (11.5%)	1 (7.1%)	0.53
Yes	54 (88.5%)	13 (92.9%)	
Boost irradiation			
Yes	13 (21.3%)	1 (7.1%)	0.2
No	48 (78.7%)	13 (92.9%)	

Data expressed as frequency (percentage). P value was significant if < 0.05. BMI, body mass index; DM, diabetes mellitus; HTN, hypertension, RTL, radiotherapy

## Discussion

Here, we prospectively investigated 75 postmenopausal early breast cancer patients undergoing adjuvant RT after surgery with either sequential or concurrent letrozole therapy. We analyzed the predictive risk factors that may affect radiation toxicity of the skin, Heart and lung. In the present study, Trastuzumab administration was fond to decrease the incidence of acute dermatitis this was agreed with De Langhe et al., (2014). which reported lower rate of acute dermatitis when trastuzumab used concurrently with radiation. It is in contradiction with the observation of Halyard et al., (2009) who reported no difference in toxicity with trastuzumab administration concurrently with adjuvant radiotherapy.

Our data support that the incidence of acute skin toxicity was significantly higher in patients receiving boost irradiation, but this is not in agreement of the results of the study done by Borm et al., (2018) who stated that boost RT is not a predictor for the increase of acute skin toxicity. Other predictive factors were found to influence radiation induced skin toxicity like bra cup size, and regular bra wearing as reported by Thongkhao et al., (2019) that regular bra-wearing during radiotherapy, in comparison to non-bra-wearing, resulted in significantly lower rates of ≥ G2 acute skin toxicity. The results of Malekzadeh et al., (2017) suggest that usage of Achillea millefolium, especially at lower doses of radiation, might decrease radiation induced dermatitis. BMI, smoking and large breast volume as detected in some studies (De Langhe et al., 2014; Freedman et al., 2006; Goldsmith et al., 2011). In the current study multivariate analysis done for late cardiac toxicity in association to demographic data and showed that hypertension was not a predictor for late cardiac toxicity. These results were in agreement with results of a study published by Khan et al., (2014) who demonstrated that the risk of ≥ grade 2 cardiotoxicity was associated with smoking, BMI ≥ 25 and left sided RT but not hypertension, chemotherapy or hormonal therapy. We also conducted an analysis of risk factors for radiation-induced pulmonary toxicities of breast cancer patients, but no significant demographic or therapy-related factors detected. Some studies reported that age and doses of chemotherapy drugs were risk factors for acute radiation lung injury (Vujaskovic et al., 2002; Ozturk et al., 2004). Matzinger et al., (2010) compared the incidence of acute radiation lung injury between internal mammary lymph nodes (IMNs) irradiation plus supraclavicular region irradiation and IMNs irradiation only and concluded that the incidence was 4.3% and 1.3%, respectively.

Wang et al., (2017) published that hypertension is an independent risk factor for acute lung toxicity and trastuzumab use is a risk factor for late radiation lung injury.

In conclusion, this study concluded that trastuzumab use decreases the incidence of acute dermatitis but boost irradiation increases it. Breast cancer patients that survive for a long time should be monitored for risk factors of their treatment schedule.
